# Changes in Parameters Registered by Innovative Technologies in Cows with Subclinical Acidosis

**DOI:** 10.3390/ani14131883

**Published:** 2024-06-26

**Authors:** Ramūnas Antanaitis, Karina Džermeikaitė, Justina Krištolaitytė, Rolandas Stankevičius, Gintaras Daunoras, Mindaugas Televičius, Dovilė Malašauskienė, John Cook, Lorenzo Viora

**Affiliations:** 1 Large Animal Clinic, Veterinary Academy, Lithuanian University of Health Sciences, Tilžės Str. 18, LT-47181 Kaunas, Lithuania; karina.dzermeikaite@lsmu.lt (K.D.); justina.kristolaityte@lsmu.lt (J.K.); mindaugas.televicius@lsmu.lt (M.T.); dovile.malasauskiene@lsmu.lt (D.M.); 2 Department of Animal Nutrition, Lithuanian University of Health Sciences, Tilzes Str. 18, LT-47181 Kaunas, Lithuania; rolandas.stankevicius@lsmuni.lt; 3 L. Kriaučeliūnas Small Animal Clinic, Veterinary Faculty, Lithuanian University of Health Sciences, LT-47181 Kaunas, Lithuania; gintaras.daunoras@lsmu.lt; 4 RCVS Recognised Specialist Cattle Health and Production, Technical Veterinarian, Avenida de los Robles Visalia, Visalia, CA 93291, USA; 2456765c@student.gla.ac.uk; 5 Scottish Centre for Production Animal Health and Food Safety, School of Biodiversity, One Health and Veterinary Medicine, University of Glasgow, Glasgow G12 8QQ, UK; lorenzo.viora@glasgow.ac.uk

**Keywords:** subclinical acidosis, innovative technologies, biomarker, dairy cows

## Abstract

**Simple Summary:**

Innovative technologies (ITs) involve real-time monitoring systems that use sensor technology to focus on individual animals. These advancements represent an important avenue for value creation across various stakeholders, with farmers primarily benefiting from its utility as a versatile tool. Our hypothesis suggested changes in biomarkers detected by innovative technologies among cows with subclinical acidosis compared to healthy ones. The results indicated that subclinical acidotic cows exhibited significantly higher activity levels compared to healthy cows, along with lower reticulorumen pH, decreased milk yield, a lower milk fat-to-protein ratio, and reduced rumination time.

**Abstract:**

The hypothesis of this study was that there were changes in biomarkers registered by innovative technologies in cows with subclinical acidosis. The aim of this study was to identify changes in the in-line milk fat-to-protein ratio and cow feeding behaviors such as reticulorumen pH, reticulorumen temperature, cow activity, and water intake with subclinical acidosis. From a total of 98 cows, 59 cows were selected to meet the following criteria (2 or more lactations, with 31 days in milk (DIM)). The selected animals were separated into two groups based on general clinical examination and reticulorumen pH: the subclinical acidosis group (SCA, *n* = 23) and the healthy group (HC, *n* = 36). During the diagnosis of subclinical acidosis and following the clinical examination of the healthy group using the BROLIS HerdLine system, the daily averages of milk yield (kg/day), milk fat (%), milk protein (%), and the milk fat-to-protein ratio were recorded. Simultaneously, by using Smaxtec technology, reticulorumen parameters and cow activity, including pH, temperature (°C), rumination time (minutes/day), and water intake (hours/day), were registered. Changes in parameters measured using innovative technologies were able to identify cows with subclinical acidosis. Cows with subclinical acidosis had a lower reticulorumen pH by 18.8% (*p* < 0.0001), a decreased milk yield by 10.49% (*p* < 0.001), a lower milk fat-to-protein ratio by 11.88% (*p* < 0.01), and a decreased rumination time by 6.59% (*p* < 0.01). However, the activity of these cows was higher by 57.19% (*p* < 0.001) compared to healthy cows. From a practical point of view, we suggest that veterinarians and farmers track parameters such as reticulorumen pH, milk yield, milk fat-to-protein ratio, rumination time, and activity for the identification of subclinical acidosis.

## 1. Introduction

High milk output is common in modern dairy farming, which poses a risk to cows’ health due to a relationship that is likely driven by survivor bias. While the observation is frequently made at the level of individual cows, it is not commonly noticed at the level of the entire herd [1]. The aim of feeding diets that are high in starch but low in fiber is to increase energy intake and meet the cow’s nutrient requirements while adhering to the limitations of the cow’s dry matter intake (DMI). Optimal milk production necessitates abundant lactose precursors, which in turn rely on glucose as a substrate for lactose synthesis. Therefore, maintaining enough glucose levels is crucial for supporting the formation of lactose [2,3]. While high starch diets are essential, they also heighten the likelihood of developing subacute ruminal acidosis (SARA), as evidenced by research [4,5,6]. SARA is a widely acknowledged digestive issue in high-producing dairy cows, detrimentally affecting both animal welfare and the economic efficiency of dairy farms, especially in well-run operations, according to previous studies [7,8,9,10]. Although cows are naturally inclined to consume forage-based diets, their nutritional intake frequently deviates. This typically results in the consumption of diets that contain a mixture of easily fermentable substrates, such as grains, or an excessive amount of long or inedible particles in the feed. This might result in alterations to one’s diet, disruptions to regular eating habits, and sporadic instances of excessive food consumption [11]. Definitions and ruminal pH benchmarks for diagnosing SARA vary across different studies; however, it typically is identified when the ruminal pH falls between 5.2 and 6 for an extended time frame [12]. SARA manifests through daily periods of reduced ruminal pH, with the pH significantly dropping for several hours each day [7,13,14], caused by an accumulation of volatile fatty acids and a lack of adequate buffering in the rumen [7]. Cows affected by SARA do not exhibit specific clinical symptoms of illness [6,15]. Nevertheless, SARA has been linked to inflammation in various organs and tissues within dairy cows. Its effects are varied and multifaceted, leading to decreased feed consumption, further exacerbating erratic eating patterns, lower digestibility of the diet, diminished milk production, a drop in the percentage of milk fat, gastrointestinal injuries, liver abscesses, and lameness [6,7]. Damage to the lining of the gastrointestinal tract, resulting in localized or systemic inflammation, is believed to be a key factor behind many of these adverse outcomes.

Innovative technologies (ITs) involve the use of real-time monitoring technologies, essentially using sensor technology to target individual animals. ITs are an important source of value creation for a range of stakeholders, although they are mostly useful for farmers as tools that offer the potential for enhancing and optimizing management in various areas, including nutrition, housing, and more. In addition to lessening environmental effects, it improves the ability to improve animal welfare, effectiveness, and health [16]. Notable real-time monitoring systems in precision livestock farming (PLF) are the BROLIS HerdLine in-line milk analyzer and the Smaxtec system, which register parameters such as reticulorumen pH and temperature. Reticulorumen temperature is a useful indicator of cattle health when used in conjunction with water intake measurements in dairy herds [17]. Real-time monitoring of reticulorumen pH and temperature is advantageous for evaluating the risk of SARA, especially in high-risk groups of cattle, such as cows in early lactation [17]. In order to forecast the nutritional and health state of dairy cows, Alzahal et al. [18] evaluated the relationship between the pH and ruminal temperature of the animals. SARA is defined as a condition in which the ruminal pH drops below 5.6 for a prolonged period. Additionally, cows with a pH lower than 5.6 had higher ruminal temperatures [18]. 

Livestock reproductive health can also be predicted using reticuloruminal pH data [9]. Low pH, or altered rumen metabolism, is associated with lower reproductive rates in dairy cows. Reticuloruminal pH is, therefore, a highly reliable indicator of a dairy cow’s procreative performance. Further research is necessary to fully understand how reticuloruminal pH affects cow reproductive health [9]. According to previous research, ruminal temperature decreases one day before parturition [19]. 

The ideal ruminal pH range for diet fermentation and fiber digestion is 6.0–6.4. Fiber is effectively broken down by the cellulolytic bacteria at this previously mentioned pH range, as it is blocked at pH values lower than 6.0 [18]. As a result, an increase in acidity due to a drop in ruminal pH raises abomasal temperature [9]. Thus, one can forecast a cow’s health state using the two criteria. Nevertheless, it is crucial to verify any findings by testing them on several management systems to guarantee that they are accurate.

Regular milking sessions, which involve the frequent and non-invasive acquisition of milk, along with established standard analysis methods, highlight the diagnostic utility of milk [20]. Precision cow husbandry has advanced due to the recent commercial availability of behavior recording systems. To improve early warning systems, more research into the application of early milk samples and the possible integration of other data sources is necessary. Before the model is put into operation, further data from other farms must be incorporated and assessed in order to guarantee that it is applicable to a variety of management styles and dietary requirements [21]. A BROLIS HerdLine in-line milk analyzer (Brolis Sensor Technology, Vilnius, Lithuania) can be used to measure the daily milk fat-to-protein ratios of cows [22]. Understanding the relationships between these features in food, blood, and milk can assist in understanding the health and production status of animals. Milk volume and components are generated from blood and food components [23]. The percentages of milk fat and protein, as well as the milk fat-to-protein (F/P) ratio, are examples of possible measurements [24]. Because milk properties match markers of lipolysis and ketogenesis in cow blood, they are an important sign of metabolic stress in cows. Since milk samples may be obtained non-invasively, they are appropriate for normal practice assessments of metabolic status. We hypothesize this would be a useful addition to herd health programs for dairy farms, and producers can identify which cows are susceptible to metabolic stress by tracking each cow’s energy status [22]. Previous research has shown that the in-line F/P ratio has a substantial positive correlation with blood non-esterified fatty acid levels, suggesting that it can be used to identify cows at increased risk of negative energy balance [22]. 

The hypothesis of this study was that subclinical acidosis in cows impacts the in-line milk fat-to-protein ratio, reticulorumen pH, reticulorumen temperature, cow activity, and water intake, as registered by innovative technologies. The aim of this study was to identify changes in the in-line milk fat-to-protein ratio and cow feeding behaviors such as reticulorumen pH, reticulorumen temperature, cow activity, and water intake with subclinical acidosis. 

## 2. Materials and Methods

### 2.1. Farm and Animals

This study adhered to the guidelines set forth by the Lithuanian Law on Animal Welfare and Protection, with an approval number of PK012858. Conducted in Lithuania, the research spanned from 1 October to 30 November 2023. Dairy cows were housed in free-stall barns equipped with ventilation systems and received a balanced total mixed ration (TMR) tailored to their physiological requirements. Feeding times were 06:00 and 18:00 daily. The cows were fed from a clean bunk. Any leftover feed was cleaned out every day at 5:00. This diet comprised 25% corn silage, 5% alfalfa grass hay, 20% grass silage, 15% sugar beet pulp silage, 30% grain concentrate mash, and 5% mineral mix, aiming to satisfy the nutritional demands of a 500 kg Holstein cow producing 37 kg of milk daily. Table 1 presents the nutritional content of the diet provided to dairy cows. The average bunk space per cow was approximately 65 cm. Milking was carried out twice daily at 05:00 and 17:00 in a parlor system. Of the 1160 cows examined, 59 were selected for this study, focusing on those in their second or subsequent lactations and within the first 5 to 30 days post-calving. The average body weight of these cows was 650 kg ± 45 kg, and they achieved an average energy-corrected milk yield (with 4.2% fat and 3.6% protein) of 12,500 kg per lactation. The calculations were performed based on predetermined formulas [25].

### 2.2. Allocation to Groups for Analysis

The 59 selected animals were separated into two groups based on general clinical examination and reticulorumen pH: the subclinical acidosis group (SCA, *n* = 23) and the healthy cow group (HC, *n* = 36).

The SCA group was created based on signs of subclinical acidosis, such as rumen motility rates of five to six times every three minutes, moderate to severe diarrhea, and the presence of undigested food particles in their feces. To analyze fiber content, fecal matter was passed through a sieve [26]. Based on the reticulorumen pH test, the pH level of the reticulorumen was found to be below 6.22 [27], and the milk fat-to-protein ratio was less than 1.2 [26].

The healthy cow group (HC) was established based on similar principles, showing ruminal motility every three minutes, no sign of diarrhea, and no presence of undigested particles in their feces. The reticulorumen pH of this group was greater than 6.22 [27].

Throughout the entire period of investigation, all cows remained within their assigned group.

### 2.3. Measurement of Variables

#### 2.3.1. Registration of In-Line Milk Fat-to-Protein Ratio

The composition of milk, including its fat and protein content, was analyzed using the BROLIS HerdLine in-line milk analyzer from Brolis Sensor Technology in Vilnius, Lithuania. This device features a GaSb widely tunable external cavity laser-based spectrometer that operates in the 2100–2400 nm spectral range and monitors milk flow in transmission mode throughout the milking process. By analyzing molecular absorption spectra, it determines the concentrations of the milk’s primary components, effectively serving as a compact, on-farm laboratory. The analyzer provides continuous assessments of each cow’s milk composition during milking. This streamlined “mini-spectroscope” is conveniently placed in the milking stalls or attached to the milking robot along the milk line. 

Each BROLIS HerdLine in-line milk analyzer was calibrated, and its accuracy was evaluated in the Eurofins lab. The resulting values of the root mean square error of prediction (RMSEP) were 0.21% for fat, 0.19% for protein, and 0.19% for lactose.

#### 2.3.2. Recticulorumen Data Collection

Reticulorumen parameters (temperature (RT), pH, and total reticulated rumination (TRR)) and physical activity were monitored using SmaXtec boluses developed by SmaXtec animal care technology^®^ (Graz, Austria), with a focus on animal health and welfare. This technology facilitates the continuous, real-time monitoring of data such as ruminal pH and temperature. The boluses, prepared as per the manufacturer’s guidelines, were administered into the cows’ reticulorumen using a specialized tool. Every cow was administered a bolus by the same skilled veterinarian. Each cow received a single bolus orally; the boluses were designed to settle in the reticulum due to gravity. Before deployment, the boluses were activated, linked to each cow’s individual ear tag for identification, and connected to the central monitoring station. The device’s functionality was verified, and it was calibrated using a standard pH 7.00 buffer solution (provided by Reagecon, Shannon, Ireland). During the administration process, cows were secured in a self-locking stand, and the individual administering the bolus manually restrained each cow’s head, opened its mouth, and used an appropriate applicator to place the bolus at the base of the tongue for the cow to swallow willingly. Post-administration, cows were observed for two hours to ensure that there were no adverse effects.

Data collection was facilitated by antennas from smaXtec animal care technology^®^, utilizing an implanted and wireless device that recorded reticulorumen temperature (RT), pH, total reticulated rumination (TRR), and physical activity. The system, managed by a microprocessor, collected pH and TRR data through an A/D converter, storing it on an external memory chip for later analysis. The smaXtec messenger^®^ software (version 4) compiled all the collected data.

Throughout the experiment, we measured several parameters: reticulorumen pH, reticulorumen temperature, cow activity, and water intake. 

### 2.4. Duration of Experimental Observation

During the diagnosis of subclinical acidosis and following the clinical examination of the healthy group using the BROLIS HerdLine system, the daily averages of milk yield (kg/day), milk fat (%), milk protein (%), and the milk fat-to-protein ratio were recorded. Simultaneously, by using Smaxtec Techmologie, reticulorumen parameters and cow activity, including pH, temperature (°C), rumination time (min/day), and water intake (h/day), were registered. 

### 2.5. Statistical Analyses 

All statistical analyses were conducted using version 25.0 of IBM SPSS Statistics for Windows (SPSS Inc., IBM Corp., Armonk, New York, NY, USA, 2017). To assess the normality of the data distribution, the Shapiro–Wilk test was utilized. Data were presented as the mean plus/minus the standard error of the mean (M ± SEM). Student’s *t*-test was applied to compare the average values of the SCK, SCA, and H groups, which were normally distributed. A *p*-value of less than 0.05 was considered statistically significant (*p* < 0.05). Pearson’s correlation coefficient was calculated to identify the linear relationship between variables. Linear regression was used to explore the statistical association between the in-line milk fat-to-protein ratio and other parameters, with significance determined at a probability of less than 0.05. All pH values were converted to H^+^ ion concentrations before running the statistics. The mean H^+^ ion concentration was calculated and then converted to pH. The SEM was presented as the H^+^ ion concentration.

## 3. Results

### 3.1. Statistical Overview of the Examined Indicators or Descriptive Distribution of Variables

We found significant differences between the groups in reticulorumen pH, milk yield, milk fat, protein, milk fat-to-protein ratio, cow activity, and rumination time (Table 2). We found that cows in the SCA group were less productive, had lower reticulorumen pH and lower levels of milk fat, protein, and milk fat-to-protein ratio (F/P), exhibited shorter rumination times, and were more active compared with the HC group.

The mean reticulorumen pH of the SCA group was 5.28 (±0.11), while in the HC group, it was 6.22 (±0.33). The reticulorumen pH in the SCA group was 18.8% lower than that in the HC group (*p* < 0.001).

Milk yield (MY) in cows from the SCA group was 10.49% lower than in cows from the HC group (*p* < 0.001). The average MY in the SCA group was 29.43 (±1.6) kg/d., while in the HC group, it was 32.52 (±1.35) kg/d. 

The milk fat-to-protein ratio (F/P) in SCA cows was 11.88% lower than in the HC group (*p* < 0.01). The average F/P ratio in the SCA group was 1.01 (±0.06), while in the HC group, it was 1.13 (±0.21).

Milk fat was 17.71% lower in the SCA group (*p* < 0.001). The average milk fat content in the SCA group was 3.67 (±0.25)%, while in the HC group, it was 4.32 (±0.81)%. 

Milk protein was also 4.95% lower in the SCA group (*p* < 0.001). The average milk protein content in the SCA group was 3.63 (±0.05)%, while in the HC group, it was 3.81 (±0.12)%. 

Cow daily activity in the SCA group was 57.19% higher than in the HC group (*p* < 0.001). The average activity of cows in the SCA group was 4.59 (±1.01), while in the HC group, it was 2.92 (±0.97).

The rumination time (RT) in the SCA group was 6.59% lower than in the HC group (*p* < 0.01). The average RT in the SCA group was 488.31 (±32.51) min/d., while in the HC group, it was 520.51 (±47.35) min/d. 

### 3.2. Correlations between Milk Fat-to-Protein Ratio and Other Variables

We found a significant association between the milk fat-to-protein ratio and reticulorumen pH, milk yield (MY), cow activity, and water intake (Table 3).

**Figure 1:**
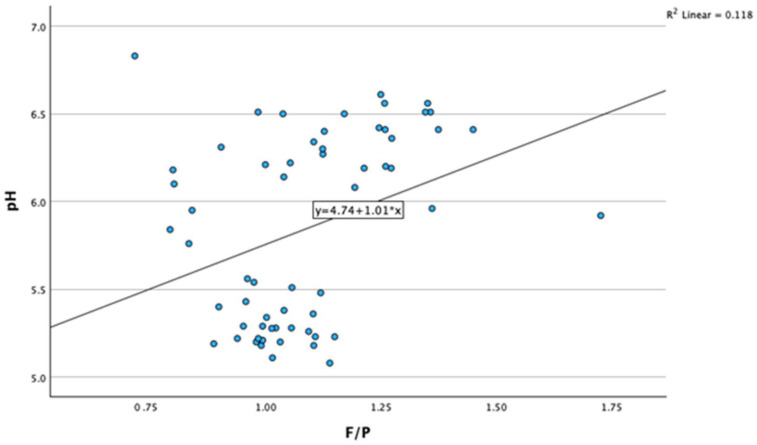
Association between reticulorumen pH and milk fat-to-protein ratio. F/P—milk fat-to-protein ratio.

We found a positive association between the fat-to-protein ratio (F/P) and reticulorumen pH (r = 0.344, *p* < 0.001). As reticulorumen pH increases, the milk fat-to-protein ratio also increases. (Figure 1).

**Figure 2:**
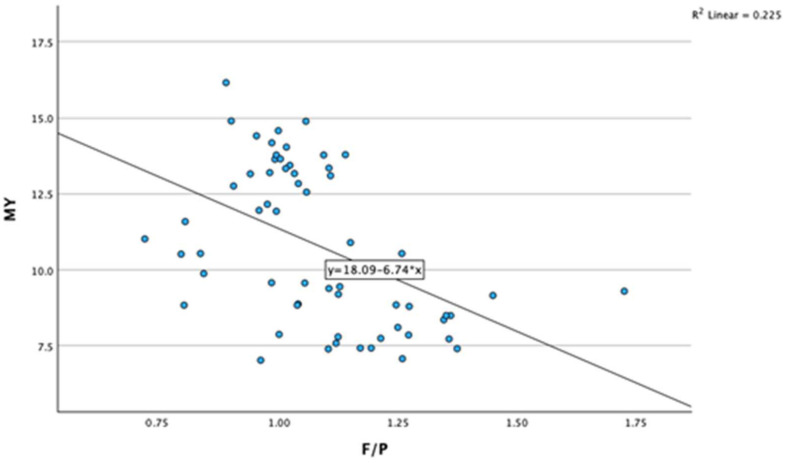
Association between milk yield and milk fat-to-protein ratio. MY—milk yield; F/P—milk fat-to-protein ratio.

A negative association was found between the fat-to-protein ratio (F/P) and milk yield (r = −0.474, *p* < 0.001). As milk yield increases, the milk fat-to-protein ratio decreases (Figure 2).

**Figure 3:**
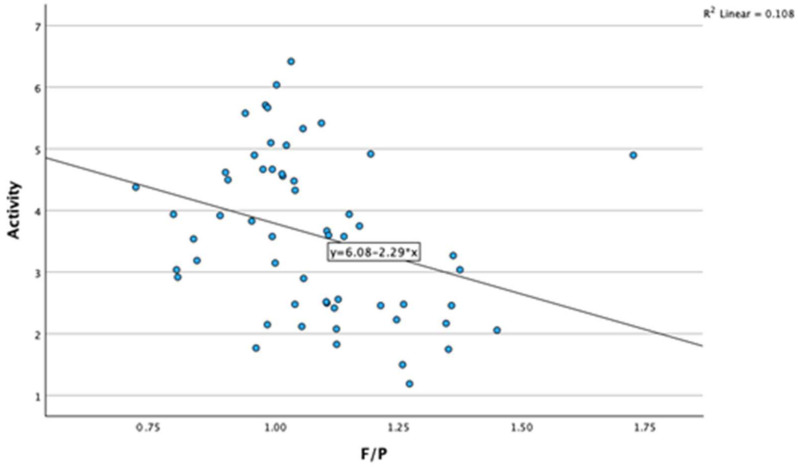
Association between cows activity and milk fat-to-protein ratio. Activity—activity of cows; F/P—milk fat-to-protein ratio.

A significant negative correlation was found between milk fat-to-protein ratio (F/P) and cow activity (r = −0.328, *p* < 0.01). As cow activity increases, the milk F/P decreases (Figure 3). 

**Figure 4:**
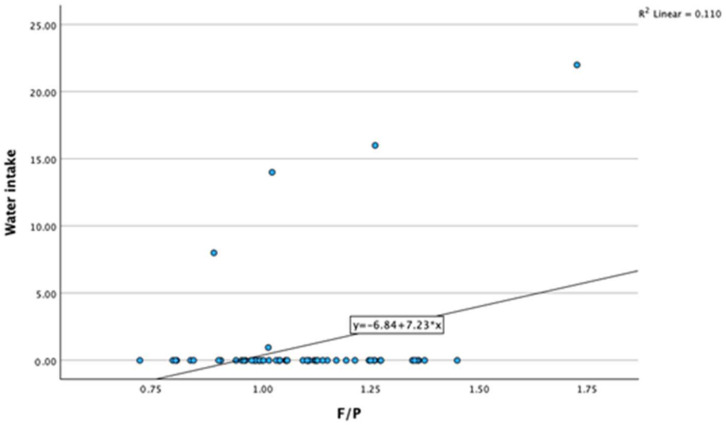
Association between water intake and milk fat-to-protein ratio. F/P—milk fat-to-protein ratio.

A significant positive correlation was found between water intake and the milk fat-to-protein ratio (F/P) (r = 0.332, *p* < 0.01). As water intake increases, the milk F/P also increases (Figure 4).

## 4. Discussion

According to our results, the activity of subclinical acidotic cows was 57.19% higher than that of healthy cows; they exhibited the following characteristics: an 18.8% lower reticulorumen pH, a 10.49% decrease in milk yield, an 11.88% lower milk fat-to-protein ratio, and a 6.59% decrease in rumination time. 

We found that cows with SCA had 18.8% lower reticulorumen pH, an 11.88% lower milk fat-to-protein ratio, and a 0.49% decrease in milk yield. A positive correlation (r = 0.344, *p* < 0.001) between the reticulorumen pH and the fat-to-protein ratio (F/P) was found. The ratio of milk fat to protein rises in tandem with an increase in reticulorumen pH. Significant correlations were discovered by Zschiesche et al. [26] between the ruminal pH and the milk F/P ratio. However, their findings indicated a negative correlation that did not align with ours. The milk F/P ratio has been identified as a valuable indicator for assessing the SCA status on a farm. Yet, additional research is required to obtain more precise findings [8]. In reality, the milk F/P ratio is often calculated in the dairy industry, and a number of studies have supported the idea that the ratio is a reliable SARA indicator (eight Holstein Friesian cows were reported to produce 25 kg of milk under trial conditions, for instance) [12]. However, other research did not find a strong enough correlation between the ruminal pH and the F/P ratio. For instance, research using 24 transition Holstein Friesian cows on a commercial farm examined the limitations of the F/P ratio as a SARA indicator [28]. 

Both a reduction in milk fat and a rise in milk protein content were necessary for changes in the F/P ratio. It has long been known that an excess in highly fermentable carbohydrates and inadequate structural effectiveness of diet are the possible causes of milk fat depression, which is characterized by a change in volatile fatty acids (VFAs) in the rumen with increased propionate and decreased acetate [26]. Sutton [29] suggested that up to 80% of the variance in milk fat might be explained by changes in the molar proportions of VFAs in the rumen. Moreover, it is currently believed that a decrease in milk fat synthesis caused by particular byproducts of ruminal fat biohydrogenation offers a somewhat convincing explanation [30]. While the association between low ruminal pH and low milk fat is well-established from a physiological perspective, the same cannot be stated for milk protein. A low pH is linked to less efficient bacterial development generally, which will have the opposite impact of the decrease in protozoa that is frequently observed with SARA diets, which will boost the efficiency of bacterial growth via reduced predation [31]. 

Conversely, as rumen volatile FA production (acetate and butyrate) acts as a precursor for mammary FA synthesis, a reduced F/P ratio may indicate (subacute) rumen acidosis [10]. Certain trials have shown indications of a relationship between the two indicators and that SARA is not solely rumen pH dependent [8,12]. In a recent exploratory meta-analysis, Mensching et al. [32] revealed milk F/P ratio as an indicator for rumen pH parameters; however, it was left out of the final prediction model. Conversely, it has been regularly noted that the F/P ratio limits in SARA prediction are significant [33]. We may infer that cows with a higher risk of subclinical acidosis can be identified using the in-line F/P ratio based on our findings and those found in the literature [22]. In previous research, we discovered that the healthy cows had the longest RT in comparison to the SARA group (4.29%) [34]. On the day of diagnosis, there were consistent decreases in rumination activity for each health condition, both within the cow and in comparison to cohorts of healthy cows [35]. In multiparous cows, rumination monitoring throughout the transition period may help detect sub-acute ketosis (SCK) and other health issues, according to Kaufman [36]. 

Cows seem to change their diurnal rumination pattern, rising during the day and decreasing during the night when they are fed large amounts of concentrates at night [33]. Rumination activity for each health issue was consistently lower on the day of diagnosis, both within the cow and when compared to cohorts of healthy mates [35]. Rumination times can be utilized to automatically detect health problems, like metritis and ketosis, that develop after calving. More research is needed to determine how RT and activity data may be used to predict the occurrence of such periparturient diseases in individuals before they occur, according to Liboreiro et al. [37]. While there may be differences in RT and activity between populations of cows that have already experienced periparturient diseases and those that were healthy, it is important to note that this association represents only one potential explanation among several. These results highlight the significant influence of SCA on feeding patterns and highlight crucial issues regarding the management of subclinical acidosis in dairy production. The productivity and well-being of the impacted cows depend heavily on the early detection and correction of such changes [38]. 

We found that cows with subclinical acidosis were 57.19% as active as healthy cows. According to Edwards and Tozer [39], there is a correlation between the symptoms used to describe a health condition and cattle activity, which typically rises prior to the appearance of clinical symptoms. Animal activity levels and variations in milk production are thought to be reliable indicators of pathological changes occurring in the body. Rising stress may be linked to an increase in animal activity prior to the discovery of an illness [40]. 

## 5. Conclusions

Changes in biomarkers measured using innovative technologies were able to identify cows with subclinical acidosis. Cows with subclinical acidosis had 18.8% lower reticulorumen pH, a 10.49% decrease in milk yield, an 11.88% lower milk fat-to-protein ratio, and a 6.59% decrease in rumination time, while the activity of these cows was 57.19% higher compared with healthy cows. From a practical point of view, we suggest that veterinarians and farmers track parameters such as reticulorumen pH, milk yield, milk fat-to-protein ratio, rumination time, and activity for the identification of subclinical acidosis. 

## Figures and Tables

**Table 1:** Chemical composition of feeding rations for dry and fresh dairy cows.

Parameters	Units	Dairy Cows
Dry matter	%	48.8
Net energy lactation	Mcal/kg	1.6
Crude protein	%	15.8
Nonfiber carbohydrates	%	38.7
Neutral detergent fiber	%	28.2
Acid detergent fiber	%	19.8

**Table 2:** Descriptive statistics of the investigated parameters.

Descriptives										
Group		N	Mean	Std. Deviation	Std. Error	95% Confidence Interval for Mean		Minimum	Maximum	*p*
						Lower Bound	Upper Bound			
pH	HC	36	6.22	0.33	6.94 × 10^−8^ M of H^+^ ion	6.11	6.33	5	7	
	SCA	23	5.28	0.11	2.42 × 10^−7^ M of H^+^ ion.	5.23	5.33	5	6	
	Total	59	5.85	0.53	1.56 × 10^−7^ M of H^+^ ion.	5.71	5.99	5	7	<0.01
DIM	HC	36	17.85	0.84	0.32	11.52	14.23	5.00	30.00	
	SCA	23	19.32	0.76	0.22	13.23	15.23	5.00	30.00	
	Total	59	154.95	17.96	2.31	150.30	159.59	126.00	227.00	0.19
MY (kg/d)	HC	36	32.52	1.35	0.22	8.54	9.46	7	13	
	SCA	23	29.43	1.60	0.32	12.66	14.01	7	16	
	Total	59	30.97	2.58	0.33	10.07	11.40	7	16	<0.01
Fat (%)	HC	36	4.32	0.81	0.13	4.05	4.60	3	6	
	SCA	23	3.67	0.25	0.05	3.57	3.78	3	4	
	Total	59	4.06	0.72	0.09	3.88	4.25	3	6	<0.01
Protein (%)	HC	36	3.81	0.12	0.02	3.77	3.85	4	4	
	SCA	23	3.63	0.05	0.01	3.60	3.65	4	4	
	Total	59	3.74	0.13	0.01	3.70	3.77	4	4	<0.01
Temperature (°C)	HC	36	38.79	1.52	0.25	38.27	39.30	33	40	
	SCA	23	38.88	1.20	0.25	38.36	39.40	35	40	
	Total	59	38.82	1.40	0.18	38.46	39.19	33	40	0.80
Cow activity	HC	36	2.92	0.97	0.16	2.59	3.25	1	5	
	SCA	23	4.59	1.01	0.21	4.15	5.03	3	6	
	Total	59	3.57	1.28	0.16	3.24	3.91	1	6	<0.01
Rumination time (min/d.)	HC	36	520.51	47.35	8.00	504.25	536.78	393	618	
	SCA	23	488.31	32.51	6.93	473.89	502.73	433	554	
	Total	59	508.09	44.81	5.93	496.20	519.98	393	618	0.01
Water_intake	HC	36	1.05	4.47	0.74	−0.45	2.56	0.00	22.00	
	SCA	23	0.95	3.29	0.68	−0.46	2.38	0.00	14.00	
	Total	59	1.01	4.02	0.52	−0.031	2.06	0.00	22.00	0.97
F/P	HC	36	1.13	0.21	0.03	1.06	1.20	0.71	1.72	
	SCA	23	1.01	0.06	0.01	0.98	1.04	0.88	1.13	
	Total	59	1.08	0.18	0.02	1.03	1.13	0.71	1.72	0.01

DIM—days in milk; MY—milk yield; min/d.—minutes per day; HC—healthy cows group; SCA—subclinical acidosis group.

**Table 3:** Correlation between milk fat-to-protein ratio and other parameters.

.		pH	DIM	MY	Fat	Protein	Temperature	Activity	RT	Water_Intake
F/P	Pearson Correlation	0.344 **	0.222	−0.474 **	0.981 **	0.167	−0.180	−0.328 *	0.110	0.332 **
	Sig. (two-tailed)	0.007	0.086	<0.001	<0.001	0.197	0.170	0.010	0.409	0.010
	N	60	61	61	61	61	60	60	58	60

** Correlation is significant at the 0.01 level (two-tailed); * Correlation is significant at the 0.05 level (two-tailed). F/P—milk fat-to-protein ratio; DIM—days in milk; MY—milk yield; fat—milk fat; protein—milk protein; temperature—reticulorumen temperature; RT—rumination time.

## Data Availability

The data provided in this study can be found in the publication.
